# Therapeutic Challenges in Metastatic Non-small Cell Lung Cancer (NSCLC) With Concurrent Mesenchymal-Epithelial Transition Factor (MET) Amplification and High Programmed Death-Ligand 1 (PD-L1) Expression: A Case Study

**DOI:** 10.7759/cureus.115338

**Published:** 2026-08-28

**Authors:** Md. Arifur Rahman, Hasin Mesbah Bin Siddique, Mohammad S Faisal, Masud Parvez, Arman Reza Chowdhury

**Affiliations:** 1 Oncology and Radiotherapy, Bangladesh Specialized Hospital PLC, Dhaka, BGD; 2 Colorectal Surgery, Bangladesh Specialized Hospital PLC, Dhaka, BGD; 3 Pathology and Laboratory Medicine, Bangladesh Specialized Hospital PLC, Dhaka, BGD; 4 Oncology, Evercare Hospital Dhaka, Dhaka, BGD

**Keywords:** capmatinib, high pd-l1, met amplification, next-generation sequencing (ngs), non-small cell lung cancer

## Abstract

Mesenchymal-epithelial transition factor (MET) amplification is an uncommon but important oncogenic driver in non-small cell lung cancer (NSCLC), and its coexistence with high programmed death-ligand 1 (PD-L1) expression creates a therapeutic dilemma over how to sequence MET-directed therapy and immune checkpoint inhibition. We report a 65-year-old man who presented with weight loss, hoarseness with left vocal cord palsy, and right supraclavicular lymphadenopathy, with metastatic disease in the lungs, lymph nodes, and bone (with hepatic and brain metastasis emerging later on therapy).

Biopsy confirmed metastatic lung adenocarcinoma. Comprehensive genomic profiling (CGP) showed MET amplification (copy number gain of 8, an intermediate-level amplification), a TP53 mutation, and high PD-L1 expression (tumor proportion score (TPS) ~80% by 22C3 immunohistochemistry at diagnosis and a VENTANA SP142 tumor cell (TC) score of 90% at progression; the two assays are not directly comparable). Because the CGP and PD-L1 results returned only after treatment had started, first-line platinum-doublet chemotherapy (nab-paclitaxel/carboplatin) was given, with a mixed response and a new hepatic metastasis. Second-line atezolizumab plus pemetrexed/carboplatin produced a partial response, followed by brain metastases. Third-line off-label capmatinib, used despite the absence of a MET exon 14 skipping mutation, produced a marked systemic response following brain radiotherapy and subsequent capmatinib. The patient then developed hepatic dysfunction that, against a background of concurrent radiological liver progression, was of uncertain cause; capmatinib was reduced and stopped, and he died approximately 30 months after diagnosis from liver failure and sepsis.

This case illustrates the difficulty of sequencing therapy when MET amplification and high PD-L1 expression coexist. Notably, a marked response to capmatinib occurred despite an intermediate MET copy number below the threshold usually associated with benefit. Any role for combined or sequential MET inhibition and immunotherapy remains a hypothesis requiring prospective study rather than a conclusion supported by this single case, and cross-assay PD-L1 results should be interpreted with caution.

## Introduction

Non-small cell lung cancer (NSCLC) is a heterogeneous disease usually diagnosed at an advanced stage, and the identification of oncogenic drivers has reshaped its treatment and improved outcomes [1]. The mesenchymal-epithelial transition factor (MET) gene encodes the hepatocyte growth factor receptor, and aberrant MET signaling drives proliferation, angiogenesis, and metastasis [2]. MET amplification, an increase in MET gene copies, can act as a primary (de novo) oncogenic driver in treatment-naïve NSCLC, the setting relevant to the present case, or arise as an acquired resistance mechanism to estimated glomerular filtration rate (EGFR)-directed therapy [2,3]. Importantly, there is no single accepted definition of MET amplification: it is variously called by the MET/CEP7 ratio on fluorescence in situ hybridization, by absolute gene copy number (GCN), and by next-generation sequencing (NGS)-derived copy number, and these methods do not agree with one another. Reported prevalence in untreated NSCLC is therefore approximately 1-5% depending on the threshold applied [4,5]. This distinction is not academic: responses to MET tyrosine kinase inhibitors (TKIs) differ sharply across low, intermediate, and high copy number strata, with the clearest benefit at high copy number (GCN ≥10) and more variable benefit at intermediate levels [5]. As detailed below, the copy number in this patient (a gain of 8) lies in the intermediate stratum, which proves central to interpreting his course.

In parallel, programmed cell death protein 1 (PD-1)/programmed death-ligand 1 (PD-L1) immune checkpoint signaling is central to NSCLC immunobiology, and PD-L1 expression on tumor cells enables immune evasion [6]. High PD-L1 expression (tumor proportion score (TPS) ≥50%) generally predicts greater benefit from PD-1/PD-L1 inhibitors [7], and several such agents are approved for advanced NSCLC, although PD-L1 alone does not fully predict response [8,9]. Critically for the present case, MET dysregulation has been associated with elevated PD-L1 expression [10], yet the coexistence of the two does not translate into reliable immunotherapy benefit: Sabari et al. reported that MET-altered lung cancers frequently express high PD-L1 but respond poorly to checkpoint blockade, with low tumor mutational burden despite high PD-L1 [7]. The prognostic impact of TP53 mutation in this setting has also been documented [11]. This is the key piece of prior evidence framing the therapeutic dilemma addressed here.

When MET amplification and high PD-L1 expression coexist in metastatic NSCLC, the optimal treatment strategy is undefined [12]. MET amplification promotes proliferation and can mediate resistance to targeted therapy, while high PD-L1 raises the question of checkpoint inhibitor use; whether the two can be exploited together is, at present, a hypothesis derived from associative data rather than a validated strategy [13]. The specific knowledge gap this report addresses is how to sequence MET-directed therapy and immunotherapy in a patient with intermediate-level MET amplification, no MET exon 14 skipping mutation, and high PD-L1 expression, a combination for which prospective evidence is lacking. We describe such a patient and the lessons his treatment course offers.

## Case presentation

A 65-year-old Bangladeshi man, with a recent 9.3-pack-year smoking history (cigarettes per day × years smoked, ÷ 20), presented in January 2022 with progressive hoarseness and weight loss. His comorbidities included hypertension, type 2 diabetes, ischemic heart disease, a prior stroke, and chronic obstructive pulmonary disease. There was no family history of lung cancer. Because he presented with hoarseness and cervical lymphadenopathy, a picture that can arise from a head-and-neck primary, a focused otorhinolaryngological (ENT) examination was performed: flexible laryngoscopy demonstrated left vocal cord palsy, and the right supraclavicular fossa contained multiple mobile, smooth nodes, the largest 1.5×1.4 cm. Positron emission tomography (PET)-computed tomography (CT) showed hypermetabolic right supraclavicular, mediastinal, hilar, and para-tracheal nodes; a focal left lower lobe pulmonary nodule; extensive abdominal nodal disease (gastrohepatic, retropancreatic, paraaortic, and paravertebral to L5); and osseous metastases, for which denosumab was subsequently started. The liver was not involved at baseline; a hepatic (segment VIII) lesion appeared only later, on post-first-line restaging (see below). Overall, this represented stage IV disease involving the lung, lymph nodes, and bone. Before histology, the differential diagnosis for bulky nodal disease with a small pulmonary nodule included lymphoma, metastatic carcinoma from a non-pulmonary primary (including a head-and-neck primary, given the laryngeal symptoms), and primary lung cancer; the diagnosis was established histologically rather than radiologically. At baseline, serum carcinoembryonic antigen (CEA) was elevated at 80.32 ng/mL, and carbohydrate antigen 19-9 (CA19-9) was normal at 1.2 U/mL (both in January 2022).

Core biopsy of the right axillary lymph node was confirmed by immunohistochemistry (IHC) as metastatic adenocarcinoma. An epiglottic biopsy showed only chronic nonspecific inflammation, with no dysplasia or malignancy, helping to exclude a laryngeal primary. IHC of the lymph node was positive for thyroid transcription factor-1 (TTF-1) and Novel Aspartic Proteinase of the Pepsin Family A (Napsin-A) and negative for p63 and synaptophysin, confirming a lung adenocarcinoma primary and excluding squamous and neuroendocrine differentiation. Comprehensive genomic profiling (CGP) established a diagnosis of NSCLC without EGFR, ALK, ROS1, BRAF, or MET exon 14 alterations, but with MET amplification with a copy number gain of 8, a TP53 mutation. PD-L1 expression at diagnosis was high, with a TPS of approximately 80% by the 22C3 IHC assay. Notably, a copy number gain of 8 constitutes intermediate-level MET amplification, below the GCN ≥10 threshold most consistently associated with MET TKI benefit, a point returned to in the Discussion.

The May 2023 re-biopsy (right cervical node) was reported as metastatic poorly differentiated carcinoma on morphology; no repeat IHC panel was performed on that specimen. The change in descriptor from the original TTF-1/Napsin-A-positive adenocarcinoma most likely reflects sampling of a less-differentiated tumor focus rather than confirmed histological transformation, and this is acknowledged as a limitation.

Molecular testing methodology

Molecular characterization was performed on two separate occasions during the disease course, using formalin-fixed, paraffin-embedded (FFPE) tumor tissue. At the initial diagnosis, CGP was performed on the right supraclavicular lymph node specimen using the 4baseCare TARGET-ABSOLUTE NGS assay (Bengaluru, India) (reported in March 2022). This platform employed hybridization capture-based targeted NGS on an Illumina sequencing platform, with alignment to the human reference genome (GRCh37/hg19); MET amplification was determined by NGS-based copy number analysis and reported as a GCN gain of 8. The 4baseCare report explicitly noted that the National Comprehensive Cancer Network (NCCN) recommends MET TKIs for high-level amplification (gain of 10 or above) and that, because the observed gain was 8, testing by an additional platform was advised before treatment decisions, which prompted the second assay. The concurrent TP53 p.Leu111Gln mutation was present at 14% allelic burden, with a DICER1 variant of uncertain significance (p.Ser1344Leu) also detected. PD-L1 expression was assessed by IHC using the PD-L1 IHC 22C3 pharmDx assay (Dako), reported as a TPS.

Molecular testing at progression

Upon disease progression, repeat CGP was performed on a lymph node specimen using the FoundationOne CDx assay (Foundation Medicine, Inc., Boston, Massachusetts, United States; sample collected in May 2023 and reported in June 2023), an FDA-approved NGS-based in vitro diagnostic device interrogating 324 genes with sequencing to high uniform depth on an Illumina platform. MET amplification was reconfirmed on this orthogonal platform, reported categorically as MET amplification (consistent with the copy number gain of 8 on the first assay; FoundationOne CDx reports MET amplification qualitatively rather than as a discrete copy number or MET/CEP7 ratio). A FAS rearrangement (intron 1) and the same TP53 L111Q mutation were also identified, and no MET exon 14 skipping mutation was detected. PD-L1 status was concurrently re-evaluated by IHC using the VENTANA PD-L1 (SP142) assay, reported as a tumor cell (TC) score of 90% and a tumor-infiltrating immune cell (IC) score of 10%. Microsatellite status was stable (MSS), and tumor mutational burden was low (4 mutations/megabase). Amplification was defined by the NGS platform's validated copy number algorithm; the two PD-L1 results were generated on different assays with different scoring conventions (22C3 TPS versus SP142 TC score) and are not directly comparable, since SP142 characteristically understains tumor cells relative to 22C3.

Treatment history

**Figure 1:**
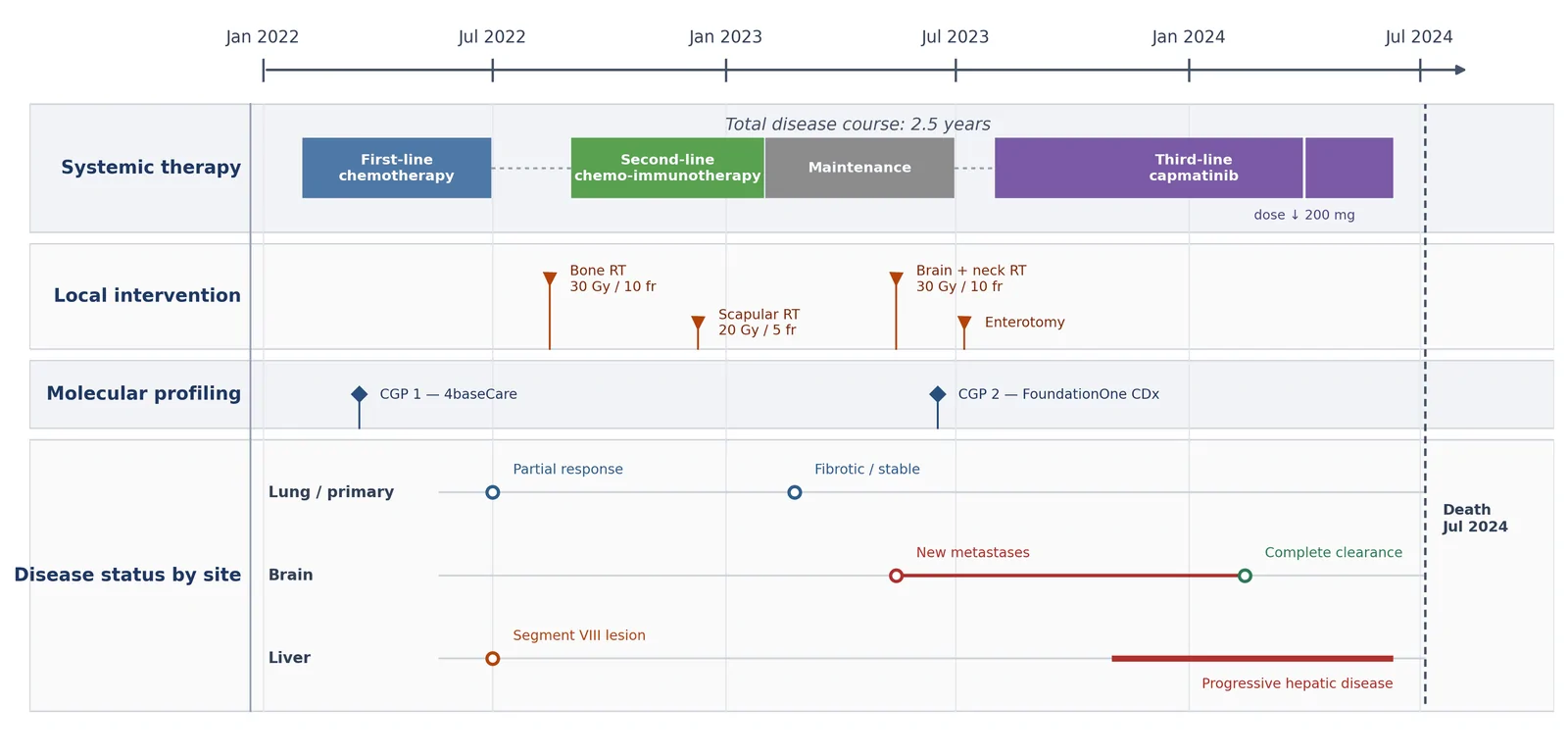
Clinical course and treatment timeline across the 2.5-year disease course Four parallel tracks are shown against a common time axis. CGP: comprehensive genomic profiling; RT: radiotherapy

**Figure 2:**
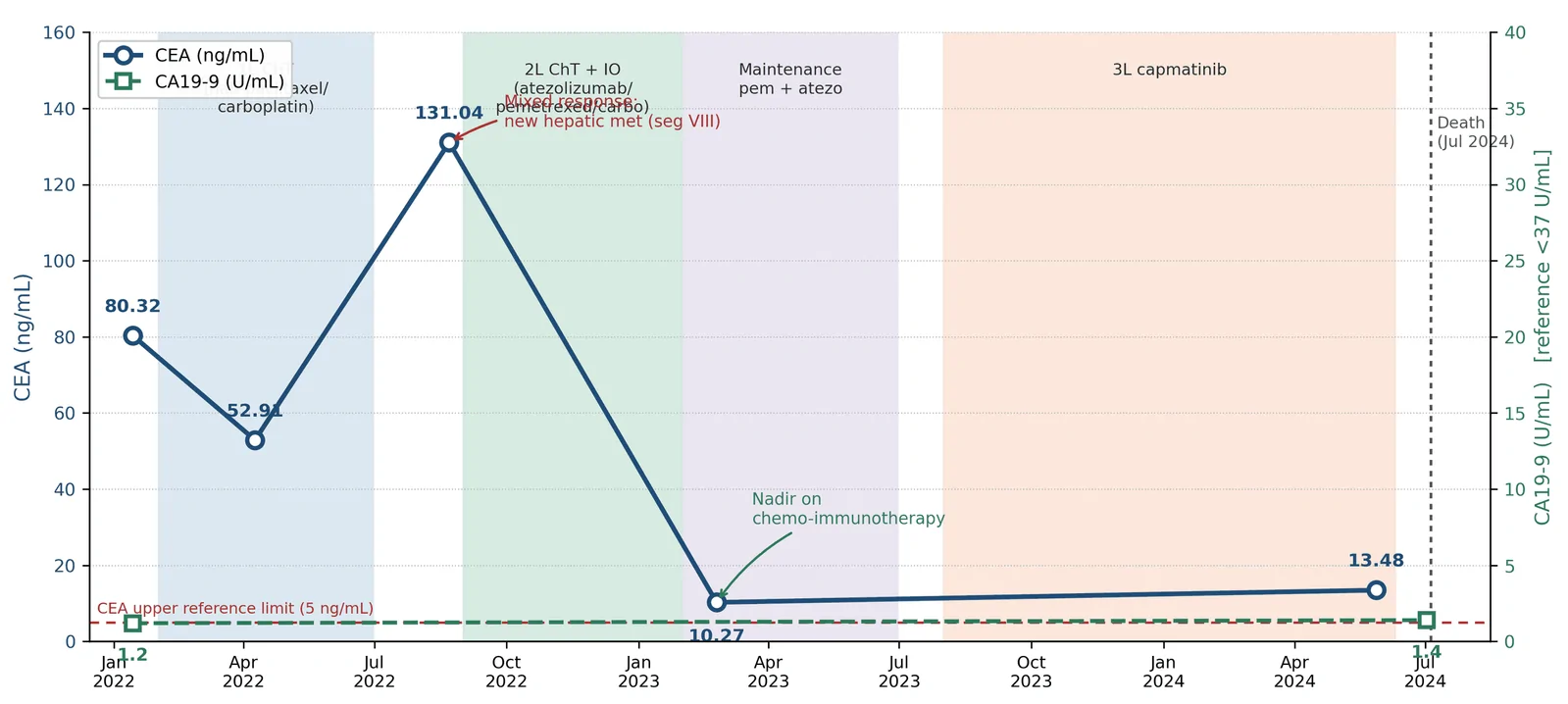
Serial tumor marker trend (CEA and CA19-9) across successive lines of therapy CEA (solid blue line, left axis) is plotted against CA19-9 (dashed green line, right axis) over the full 30-month disease course, with shaded bands denoting each treatment phase. CEA: carcinoembryonic antigen; CA19-9: carbohydrate antigen 19-9; ChT: chemotherapy; IO: immunotherapy (immune-oncology); 1L: first line; 2L: second line; 3L: third line; pem: pemetrexed; atezo: atezolizumab; carbo: carboplatin

The complete treatment course is summarized chronologically in Figure 1. Because the CGP and PD-L1 results were not reported until March 2022, after systemic therapy had already begun, the initial regimen was chosen without the benefit of the molecular profile, a biomarker-turnaround constraint relevant to resource-limited settings. Given metastatic disease and no classic driver mutation (EGFR/ALK/ROS1), first-line platinum-doublet chemotherapy was selected over upfront immunotherapy. From February 2022, the patient received six three-weekly cycles of nab-paclitaxel and carboplatin with denosumab for bone metastases, completed in June 2022. Restaging PET-CT showed a mixed metabolic response (Positron Emission Tomography Response Criteria in Solid Tumors (PERCIST)): most osseous lesions responded or stabilized, but a new hypermetabolic hepatic lesion (segment VIII) appeared, indicating overall progression. CEA fell from 80.32 ng/mL (January 2022) to 52.91 ng/mL (April 2022) but rose to 131.04 ng/mL by August 2022, coinciding with the hepatic lesion (Figure 2 and Table 1). First-line progression-free survival was approximately six months.

**Table 1:** Serial serum tumor marker values with corresponding clinical and treatment context CEA broadly paralleled disease activity during the earlier treatment course but became discordant with radiological progression later. CEA: carcinoembryonic antigen; CA19-9: carbohydrate antigen 19-9; CT: computed tomography

Timeline	CEA (ng/mL)	CA19-9 (U/mL)	Clinical and treatment context
January 2022	80.32	1.2	Baseline at diagnosis, before any systemic therapy
April 2022	52.91	-	During first-line nab-paclitaxel/carboplatin: falling
August 2022	131.04	-	After first-line chemotherapy: rising, concurrent with mixed response and new hepatic metastasis (segment VIII)
February 2023	10.27	-	Nadir after second-line atezolizumab/pemetrexed/carboplatin: paralleling partial metabolic response
May 2024	13.48	-	On third-line capmatinib, at the onset of hepatic failure: near-normal despite radiological hepatic progression
July 2024	-	1.4	Terminal phase: progression on CT of the abdomen despite normal CA19-9

In view of the high PD-L1 expression, second-line atezolizumab plus pemetrexed and carboplatin was begun in September 2022 and continued for six cycles to January 2023. Interim PET-CT showed a partial metabolic response with regression of nodal, pulmonary, and osseous disease, and CEA fell to a nadir of 10.27 ng/mL in February 2023 (a 92% reduction; Figure 2 and Table 1). During this period, palliative conventional 3DCRT radiotherapy was delivered for painful bone metastases: to the lumbar spine (L5) and pelvis (left hemipelvis) (30 Gy in 10 fractions, July 2022) and subsequently to the right scapula (3DCRT, 20 Gy in five fractions, December 2022). Maintenance pemetrexed with atezolizumab followed but was complicated by severe neutropenia and, in July 2023, by gallstone ileus causing intestinal obstruction that required surgical enterotomy with primary closure. Second-line progression-free survival was approximately eight months.

**Figure 3:**
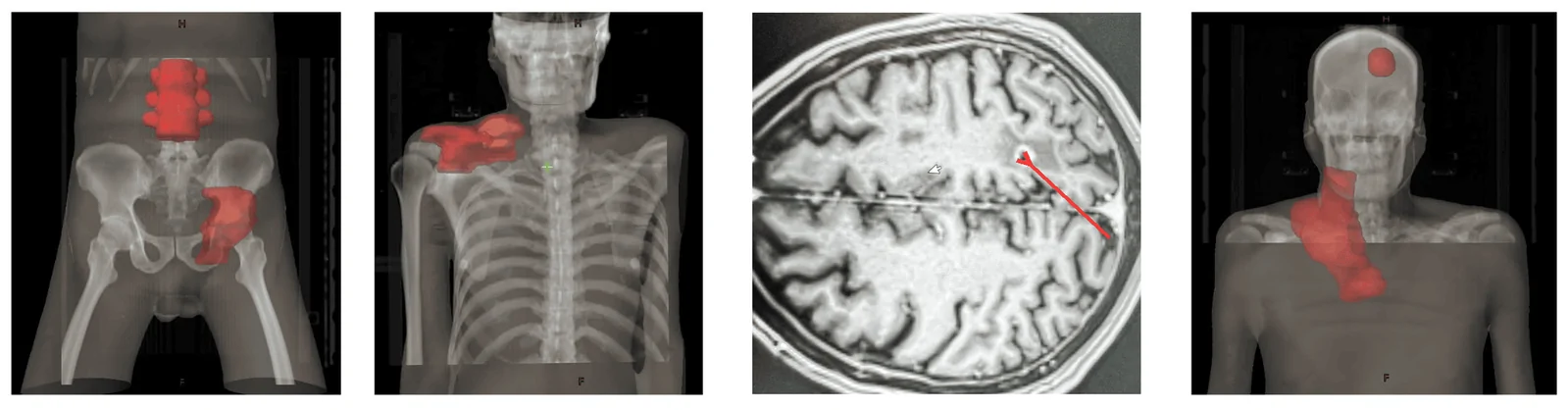
Radiotherapy treatment sites and dose/fractionation with brain (diagnostic) MRI with axial post-contrast. Composite planning images showing the three palliative radiotherapy episodes delivered over the disease course: (left) lumbar spine and left pelvis, 30 Gy in 10 fractions, July 2022; (center) right scapula, 20 Gy in five fractions, December 2022; and (right) brain and right neck/supraclavicular lymph nodes, 30 Gy in 10 fractions, May 2023. All courses used conventional fractionation for palliation and symptom control; the brain lesion was a solitary left parietal metastasis treated with focal (conformal) IMRT at 30 Gy in 10 fractions (not whole-brain radiotherapy and not stereotactic radiosurgery) Red-shaded areas indicate the planning target volumes. Red arrows show the solitary left parietal metastatic lesion. MRI: magnetic resonance imaging; IMRT: intensity-modulated radiotherapy; Gy: gray

In May 2023, contrast-enhanced magnetic resonance imaging (MRI) of the brain, the appropriate modality for central nervous system (CNS) assessment, revealed a new solitary brain metastasis (a left parietal lesion), together with progression in the right neck and scapula; the Eastern Cooperative Oncology Group (ECOG) Performance Status was 1-2. Radiotherapy was delivered to a solitary left parietal brain metastasis using focal (conformal) IMRT directed to the lesion, not whole-brain radiotherapy, and to the recurrent neck/scapular sites, each 30 Gy in 10 fractions (completed May 2023) (Figure 3). A second core biopsy from a right cervical node in May 2023 was reported as metastatic poorly differentiated carcinoma; repeat CGP (FoundationOne CDx) reconfirmed MET amplification with an elevated MET/CEP7 ratio and no MET exon 14 skipping mutation, and PD-L1 by SP142 showed a TC score of 90%.

**Figure 4:**
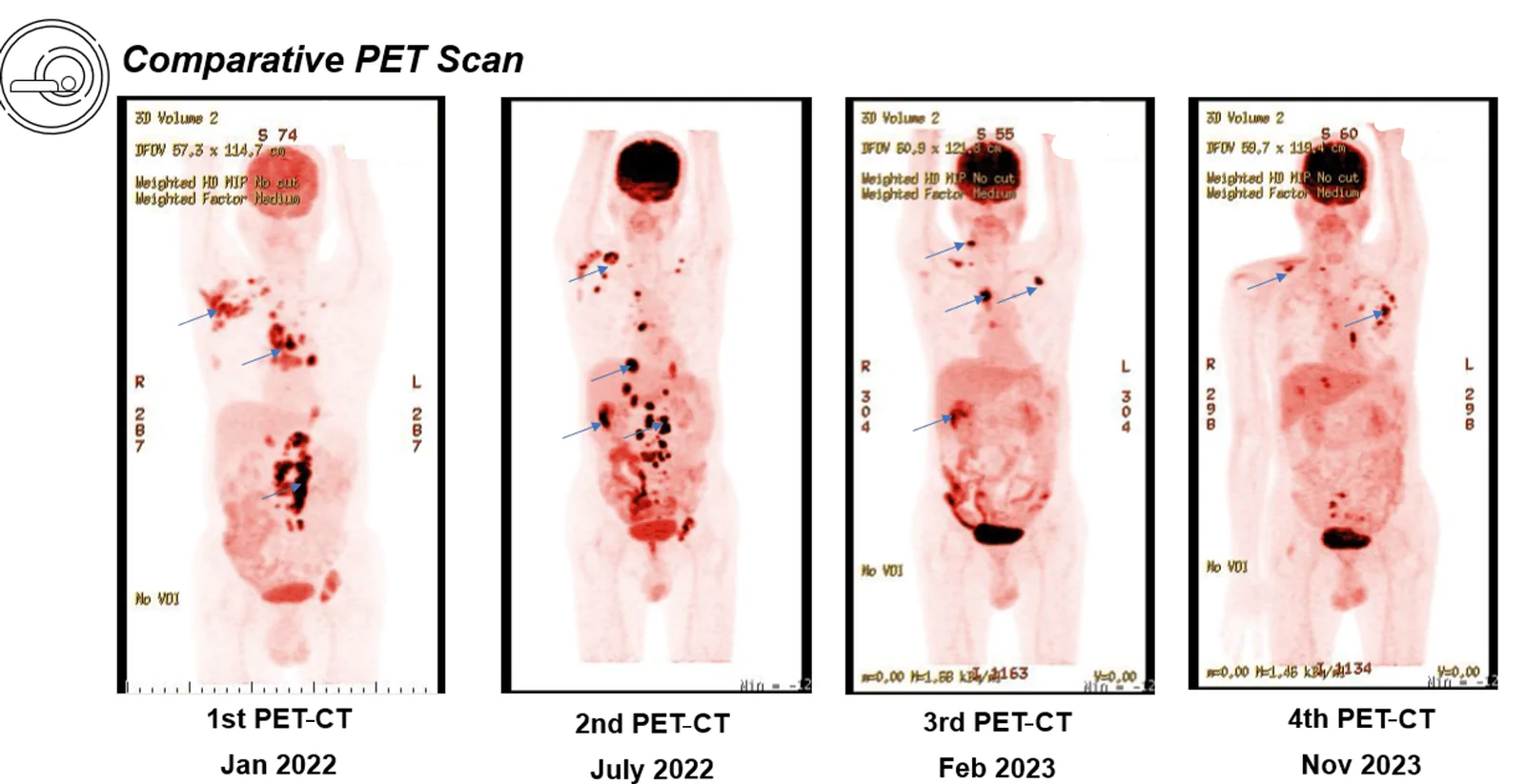
Serial comparative ¹⁸F-FDG PET-CT MIP images demonstrating the disease response trajectory with corresponding treatment timeline. Four serial whole-body PET-CT scans are shown in chronological order, each annotated with arrows identifying the key metastatic lesions and labeled with the corresponding date and treatment stage Arrows indicate the representative sites of metabolically active disease. ¹⁸F-FDG: fluorine-18 fluorodeoxyglucose; PET-CT: positron emission tomography-computed tomography; MIP: maximum intensity projection

**Figure 5:**
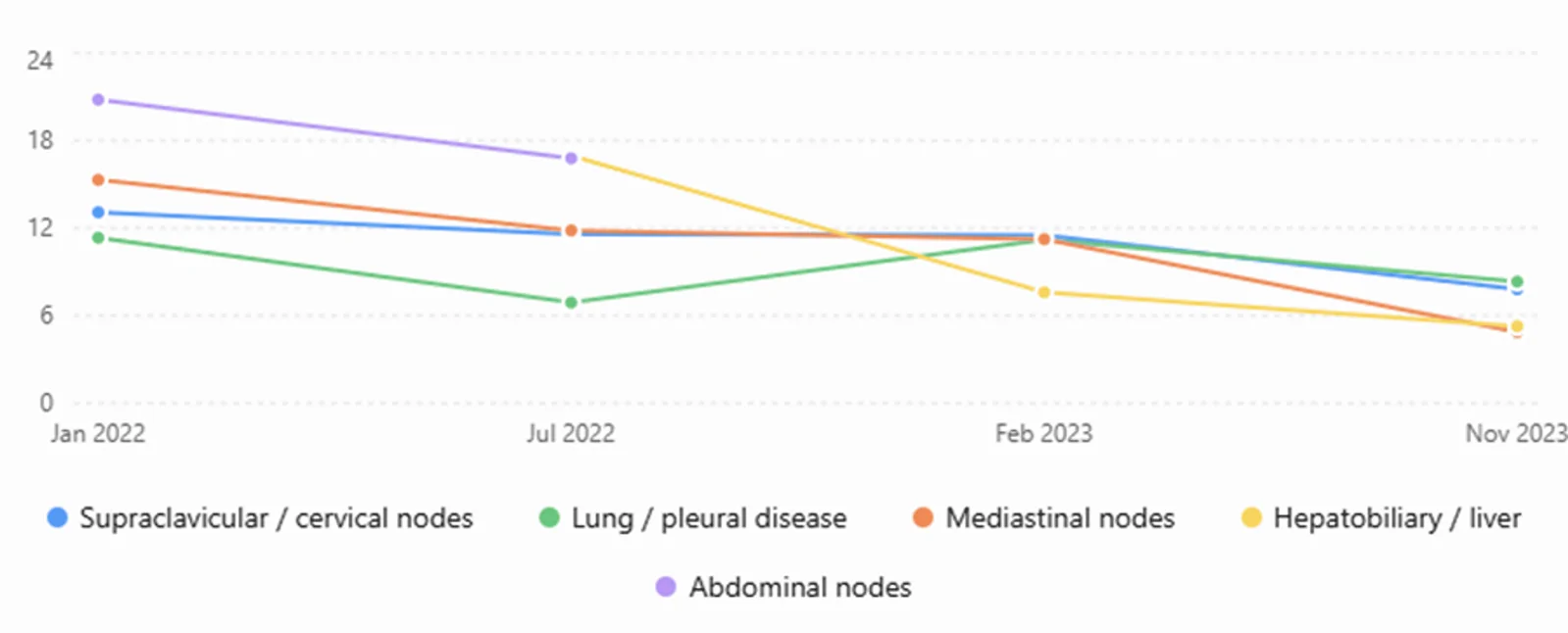
Serial ¹⁸F-FDG PET-CT metabolic response by disease compartment Highest reported SUVmax in each major disease compartment across the four PET-CT examinations (January 2022, July 2022, February 2023, November 2023). Lesions are not necessarily identical targets at each scan. The hepatobiliary curve begins only in July 2022, reflecting the absence of any hepatic lesion at baseline and the emergence of the segment VIII metastasis on first-line restaging (SUVmax 16.96), with subsequent decline. Abdominal nodal disease (peak SUVmax 20.80) resolved after second-line therapy. Across all compartments a broad metabolic decline is evident by November 2023, during the capmatinib response. ¹⁸F-FDG: fluorine-18 fluorodeoxyglucose; PET-CT: positron emission tomography-computed tomography; SUVmax: maximum standardized uptake value

Third-line self-funded capmatinib (400 mg twice daily) was started in August 2023. Capmatinib was used off-label: the patient had no MET exon 14 skipping mutation (confirmed on both assays), and capmatinib's regulatory approval is for MET exon 14 skipping rather than MET amplification. The response was marked: resolution of hoarseness, a very good partial metabolic response on serial PET-CT (dominant mediastinal nodal SUVmax 15.30 → 4.87; hepatic lesion SUVmax, absent at baseline, 16.96 at emergence → 5.28 (Figures 4-5), and, strikingly, complete intracranial clearance on MRI in February 2024 despite the intermediate copy number and prior brain radiotherapy confound attribution. Subsequently, the patient developed rising liver enzymes, jaundice, and edema. Because CT of the abdomen concurrently showed hepatic disease progression (with pleural effusion and lymphadenopathy), the hepatic dysfunction had two plausible causes, that is, drug-induced liver injury from capmatinib and progression of liver metastases, and these cannot be disentangled with the data available (see Discussion for a structured causality assessment). Liver dysfunction developed in late June 2024 (see below); capmatinib was dose-reduced to 200 mg and then discontinued as hepatic function deteriorated. Performance status declined to ECOG 3, further anti-cancer therapy was not feasible, and the patient died in July 2024, approximately 30 months after diagnosis, from liver failure and sepsis. CEA in May 2024 was 13.48 ng/mL, still more than four times the upper reference limit, yet markedly lower than the extent of radiological hepatic progression would predict, illustrating discordance between the marker and imaging; CA19-9 was normal on the two occasions measured and was not used to guide management. The interval to this confirmatory MRI was extended by an intercurrent gallstone ileus laparotomy (July 2023) and the start of third-line therapy; however, an interim restaging fluorine-18 fluorodeoxyglucose (¹⁸F-FDG) PET-CT in November 2023 had already shown no residual brain lesion, and the February 2024 MRI (reported as no metastatic deposit, with only mild generalized atrophy and bilateral microvascular white matter change) confirmed the sustained intracranial response.

## Discussion

This patient had metastatic lung adenocarcinoma with intermediate-level MET amplification (copy number gain of 8) and a TP53 mutation. The observed NGS copy number gain of 8 corresponds to the GCN 6-9 intermediate stratum used in GEOMETRY mono-1, which lies below the GCN ≥10 high-level cohort. TP53 is among the most frequently mutated genes in NSCLC, and its inactivation promotes tumor progression [14]; co-occurrence of TP53 mutation with MET amplification and with PD-L1 expression has been described [15]. The most instructive feature of this case concerns the MET copy number stratum. Capmatinib is most effective in high-level amplification (GCN ≥10), and in GEOMETRY mono-1, response rates were lower below the high-level threshold (GCN ≥10) in which this patient (copy number gain of 8) does not fall; indeed, the molecular report itself flagged this sub-threshold value and recommended orthogonal confirmation before MET-directed treatment [16]. Yet he achieved a marked systemic response and complete intracranial clearance. This apparent discordance, a strong response below the copy number threshold usually associated with benefit, is the main clinical lesson of the case and suggests that copy number alone may not fully predict MET TKI benefit in individual patients. Regarding PD-L1, the two assays used are not directly comparable (22C3 TPS ~80% at diagnosis; SP142 TC 90% at progression), so the results cannot be read as showing stable expression over time; this cross-assay limitation is acknowledged. Tumor markers were of limited value: only five CEA and two CA19-9 measurements were obtained over 30 months (with a 15-month gap during third-line therapy), so these are sparse retrospective observations rather than serial monitoring. CEA broadly tracked the earlier disease course but, at 13.48 ng/mL near the end, substantially underestimated the extent of radiological hepatic progression; CA19-9 was normal on both occasions measured. CEA and CA19-9 are not standard NSCLC response tools and did not guide management.

The choice of first-line chemotherapy over immunotherapy in a PD-L1-high tumor warrants comment. In this patient, the decision was driven by biomarker turnaround (the molecular and PD-L1 results returned only after treatment had started) rather than by a considered preference against immunotherapy, a practical constraint familiar in resource-limited settings. Carboplatin was retained in the second-line regimen given prior tolerance and the addition of atezolizumab. Mechanistically, MET encodes a receptor tyrosine kinase whose aberrant signaling promotes proliferation, invasion, and metastasis, and MET is a recognized resistance pathway in NSCLC [2,5]. High MET activity and high PD-L1 may coexist, but as Sabari et al. showed, high PD-L1 in MET-altered NSCLC does not reliably translate into checkpoint inhibitor benefit [7], consistent with this patient's limited durable benefit from immunotherapy and eventual dependence on MET-directed treatment.

Capmatinib is a selective MET inhibitor approved for NSCLC with MET exon 14 skipping; tepotinib is similarly approved for MET exon 14 skipping, while savolitinib is approved regionally (in China) rather than by the FDA [16]. Crizotinib has also shown activity specifically in MET-amplified NSCLC [17]. This patient had no MET exon 14 skipping mutation, so capmatinib was used off-label for MET amplification. In GEOMETRY mono-1, the 68% overall response rate (ORR) was observed in treatment-naïve patients with MET exon 14 skipping mutations. In MET-amplified NSCLC, activity was greatest at GCN ≥10 (ORR 40% in treatment-naïve and 29% in previously treated patients), whereas efficacy was substantially lower at GCN <10 with intermediate copy number such as ours [16]. Hepatotoxicity is a recognized capmatinib effect: in GEOMETRY mono-1, alanine aminotransferase (ALT) elevation was somewhat more frequent among immune checkpoint inhibitor (ICI)-pretreated patients (15.6% any grade) than in ICI-naïve patients (11.8%) [18], and severe capmatinib-associated hepatotoxicity has been reported [19]. Our patient had prior atezolizumab exposure, which may be relevant to his hepatic course.

Attributing the hepatic dysfunction specifically to capmatinib is not straightforward, because CT concurrently demonstrated progressive liver metastases. The serial liver chemistry (Table 2) is informative: liver enzymes were near-normal throughout the first several months of capmatinib, including in February 2024 during the peak response, and rose only in late June 2024. The terminal derangement was predominantly cholestatic (a marked rise in bilirubin (0.3 → 5.7 mg/dL, mostly direct) and alkaline phosphatase (ALP) (87 → 238 U/L) with only modest transaminase elevation (ALT ≤55 U/L; R-ratio≈0.7)) accompanied by rising international normalized ratio (INR) and falling albumin, indicating impaired synthetic function. Applying the Roussel Uclaf Causality Assessment Method (RUCAM), several features argue against classic capmatinib hepatotoxicity, which is typically hepatocellular (transaminase-predominant) and of earlier onset: the long latency (approximately 10 months), the cholestatic pattern, and above all the concurrent radiological hepatic progression as a compelling competing cause. On balance, we judge capmatinib causality to be low ("unlikely to possible"), with malignant hepatic infiltration/biliary compromise the more probable driver of the terminal liver failure. We therefore no longer attribute the hepatic failure primarily to the drug and present the two processes as concurrent.

**Table 2:** Serial liver chemistry across the disease course Values were stable and near-normal throughout chemotherapy, immunotherapy, and the first months of capmatinib (including February 2024, during the capmatinib response) and then rose sharply in late June-July 2024. The terminal pattern was predominantly cholestatic (marked rise in bilirubin and ALP with only modest transaminase elevation; R-ratio≈0.7), coinciding with CT-documented hepatic progression. CT: computed tomography; ULN: upper limit of normal (laboratory reference); ALT: alanine aminotransferase; AST: aspartate aminotransferase; ALP: alkaline phosphatase; GGT: gamma-glutamyl transferase; INR: international normalized ratio; -: not measured at that time

Analyte (ULN)	February-November 2022	July 2023	February 2024	Early June 2024	Late June 2024	July 2024
ALT, U/L (≆40)	17-39	55-62	19	50	55	-
AST, U/L (≆40)	-	-	-	-	42	-
ALP, U/L (≤120)	70-78	159-196	87	-	238	-
GGT, U/L	-	134	-	-	159	-
Bilirubin total, mg/dL	0.3	-	-	-	4.6	5.7
Bilirubin direct, mg/dL	-	-	-	-	0.9	1.3
Albumin, g/L	36.6	25-29	23.6	25-26	22.2	25.5
INR	-	1.0-1.29	-	-	1.59	1.59

Other reports illustrate the heterogeneity of MET-driven disease: Choi et al. described primary capmatinib resistance in a MET-overexpressing tumor with concurrent MYC amplification [20], and Ding et al. reported an ALK-positive NSCLC with ultra-high PD-L1 that acquired MET amplification and responded to combined lorlatinib and the PD-L1 inhibitor envafolimab [13]. Such reports raise the possibility that dual targeting of MET and the PD-1/PD-L1 axis might be beneficial in selected patients, but this remains a hypothesis generated from isolated cases and associative data. It was not tested in our patient, who received checkpoint inhibition and MET inhibition sequentially, months apart and separated by an intervening surgical complication, and no inference about combination therapy can be drawn from this course. Emerging MET-directed options, including MET-targeted antibody-drug conjugates, may broaden treatment for MET-altered NSCLC in the future.

Limitations

Several limitations apply. This is a single case, from which no generalizable efficacy or causal inference can be drawn. Capmatinib was used off-label without rechallenge, so the hepatotoxicity could not be tested by re-exposure. No tumor tissue or plasma circulating tumor DNA was obtained at progression on capmatinib, so the mechanism of eventual resistance is unknown. PD-L1 was assessed on two non-comparable assays at different timepoints, precluding conclusions about change over time. Tumor marker sampling was sparse and irregular. Finally, although serial liver chemistry allowed a structured (RUCAM) causality assessment, the concurrent hepatic progression and the absence of a drug rechallenge mean the contribution of capmatinib to the terminal liver failure cannot be fully excluded.

## Conclusions

This case documents a patient with metastatic NSCLC harboring intermediate-level MET amplification (copy number gain of 8), no MET exon 14 skipping mutation, and high PD-L1 expression, who, after limited durable benefit from chemotherapy and chemo-immunotherapy, achieved a marked systemic response and complete intracranial clearance on off-label capmatinib in the third line, before developing hepatic dysfunction of uncertain cause that ended treatment; he survived approximately 30 months with stage IV disease. The principal lesson is the difficulty of sequencing therapy when MET amplification and high PD-L1 expression coexist, compounded by real-world constraints such as biomarker turnaround and off-label access. The response below the usual copy number threshold is noteworthy and hypothesis-generating. Any role for combined or sequential MET inhibition and immunotherapy requires prospective evaluation and cannot be established from this single case.
